# Poly[bis­(μ_4_-benzene-1,4-dicarboxyl­ato)(μ_4_-succinato)diterbium(III)]

**DOI:** 10.1107/S1600536808011355

**Published:** 2008-04-30

**Authors:** Chun-Hui Yu

**Affiliations:** a Department of Chemistry, College of Chemistry and Biology, Beihua University, Jilin City 132013, People’s Republic of China

## Abstract

In the title compound, [Tb_2_(C_4_H_4_O_4_)(C_8_H_4_O_4_)_2_]_*n*_, the coord­in­ation around each Tb atom is distorted square-anti­prismatic. The benzene-1,4-dicarboxyl­ate and succinate anions bridge the anti­prisms, forming a three-dimensional network. The succinate anion is located on a centre of inversion. The structure is isomorphous with the Dy, Gd, Er and Nd complexes.

## Related literature

For isomorphous structures, see: Wang & Li (2005 ▶) He *et al.* (2006 ▶); Li & Wang (2005 ▶); Li *et al.* (2006 ▶).
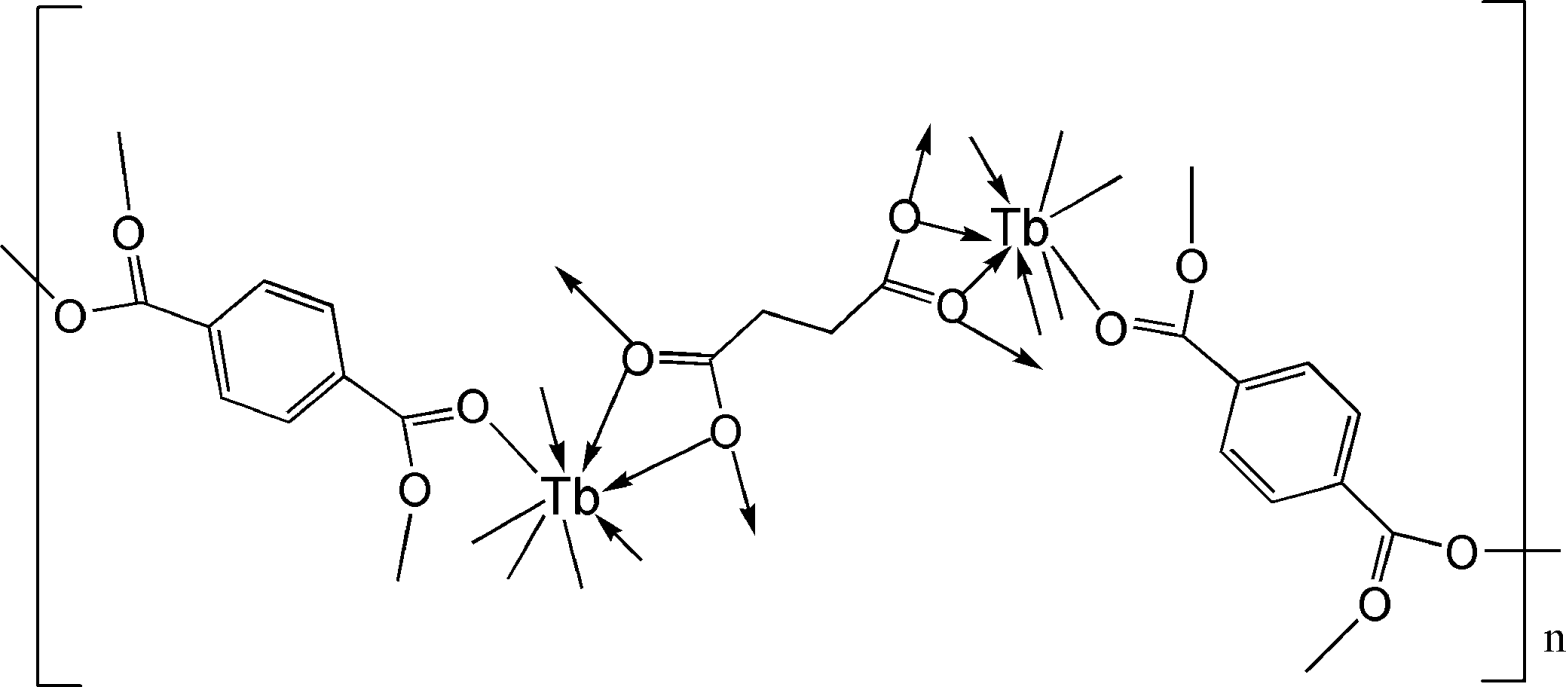


## Experimental

### 

#### Crystal data

[Tb_2_(C_4_H_4_O_4_)(C_8_H_4_O_4_)_2_]
*M* *_r_* = 381.07
Orthorhombic, 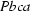

*a* = 13.948 (3) Å
*b* = 6.8724 (14) Å
*c* = 21.844 (4) Å
*V* = 2093.9 (7) Å^3^
*Z* = 8
Mo *K*α radiation
μ = 6.77 mm^−1^
*T* = 293 (2) K
0.29 × 0.27 × 0.20 mm

#### Data collection

Rigaku R-AXIS RAPID diffractometer
Absorption correction: multi-scan (*ABSCOR*; Higashi, 1995 ▶) *T*_min_ = 0.121, *T*_max_ = 0.257
18548 measured reflections
2376 independent reflections
2135 reflections with *I* > 2σ(*I*)
*R*_int_ = 0.027

#### Refinement

*R*[*F* ^2^ > 2σ(*F* ^2^)] = 0.017
*wR*(*F* ^2^) = 0.041
*S* = 1.07
2376 reflections
154 parameters
H-atom parameters constrained
Δρ_max_ = 1.09 e Å^−3^
Δρ_min_ = −0.44 e Å^−3^

### 

Data collection: *PROCESS-AUTO* (Rigaku, 1998 ▶); cell refinement: *PROCESS-AUTO*; data reduction: *PROCESS-AUTO*; program(s) used to solve structure: *SHELXS97* (Sheldrick, 2008 ▶); program(s) used to refine structure: *SHELXL97* (Sheldrick, 2008 ▶); molecular graphics: *SHELXTL-Plus* (Sheldrick, 2008 ▶); software used to prepare material for publication: *SHELXL97*.

## Supplementary Material

Crystal structure: contains datablocks global, I. DOI: 10.1107/S1600536808011355/bt2699sup1.cif

Structure factors: contains datablocks I. DOI: 10.1107/S1600536808011355/bt2699Isup2.hkl

Additional supplementary materials:  crystallographic information; 3D view; checkCIF report

## Figures and Tables

**Fig. 1.**
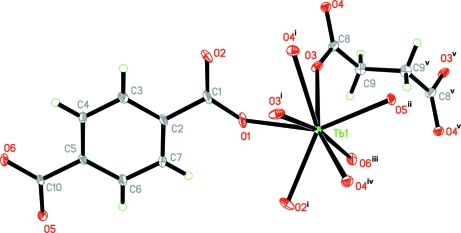
The structure of (I), showing the atomic numbering scheme. Displacement ellipsoids are drawn at the 30% probability level. Symmetry codes: (i) 0.5 - x, y - 1/2, z; (ii) 0.5 - x, -y, z + 1/2; (iii) x + 1/2, y, 0.5 - z; (iv) x, y - 1, z; (v) 1 - x, 1 - y, 1 - z.

## supplementary crystallographic information

### Comment

Lanthanide complexes usually exhibit interesting framework structures and intense luminescence. In all types of rare-earth compounds, carboxylate anions with aromatic rings are widely used in the construction of high-dimensional lanthanide coordination polymers because these anions are able to act as bridging ligands in various ligating modes. However, rare-earth coordination polymers with mixed aromatic and fatty carboxylates are rarely studied (Wang & Li, 2005). In this paper, we present a new mixed carboxylate complex, namely [Tb_2_(1,4-bdc)_2_(suc)]_n_ (I), where 1,4-bdc=benzene-1,4-dicarboxylate and suc= succinate.

In (I), each Tb(III) center is coordinated by eight O atoms from 1,4-bdc and suc anions in a distorted square antiprism (Fig. 1). Each carboxylate moiety of the 1,4-bdc bridges two Tb(III) atoms, whereas each carboxylate group of suc links four Tb(III) atoms. In these modes, the central Tb(III) atoms are linked by 1,4-bdc and suc ligands, resulting in a rare three-dimensional framework structure (Fig. 2). The succinate anion is located on a centre of inversion. The structure of the title compound is isomorphous with the Dy (Li & Wang, 2005) Gd (Wang & Li, 2005), Er (He *et al.*,  2006) and the Nd (Li *et al.*, 2006) complex.

### Experimental

A mixture of 1,4-H_2_bdc (0.5 mmol), H_2_suc (0.5 mmol), NaOH (1 mmol) and TbCl_3_^.^6H_2_O (0.5 mmol) was suspended in 12 ml of deionized water and sealed in a 20-ml Teflon-lined autoclave. Upon heating at 185°C for ten days, the autoclave was slowly cooled to room temperature. The crystals were collected, washed with deionized water and dried.

### Refinement

H atoms bonded to C atom were positioned geometrically (C—H = 0.93 Å) and refined as riding, with *U*_iso_(H)=1.2*U*_eq_(carrier).

### Crystal data

**Table:**

[Tb_2_(C_4_H_4_O_4_)(C_8_H_4_O_4_)_2_]	*F*(000) = 1432
*M**_r_* = 381.07	*D*_x_ = 2.418 Mg m^−^^3^
Orthorhombic, *P**b**c**a*	Mo *K*α radiation, λ = 0.71073 Å
Hall symbol: -P 2ac 2ab	Cell parameters from 15551 reflections
*a* = 13.948 (3) Å	θ = 3.0–27.5°
*b* = 6.8724 (14) Å	µ = 6.77 mm^−^^1^
*c* = 21.844 (4) Å	*T* = 293 K
*V* = 2093.9 (7) Å^3^	Block, colorless
*Z* = 8	0.29 × 0.27 × 0.20 mm

### Data collection

**Table:**

Rigaku R-AXIS RAPID diffractometer	2376 independent reflections
Radiation source: rotating anode	2135 reflections with *I* > 2σ(*I*)
graphite	*R*_int_ = 0.027
Detector resolution: 10.0 pixels mm^-1^	θ_max_ = 27.5°, θ_min_ = 3.4°
ω scans	*h* = −18→18
Absorption correction: multi-scan (*ABSCOR*; Higashi, 1995)	*k* = −8→8
*T*_min_ = 0.121, *T*_max_ = 0.257	*l* = −28→28
18548 measured reflections	

### Refinement

**Table:**

Refinement on *F*^2^	Primary atom site location: structure-invariant direct methods
Least-squares matrix: full	Secondary atom site location: difference Fourier map
*R*[*F*^2^ > 2σ(*F*^2^)] = 0.017	Hydrogen site location: inferred from neighbouring sites
*wR*(*F*^2^) = 0.041	H-atom parameters constrained
*S* = 1.07	*w* = 1/[σ^2^(*F*_o_^2^) + (0.0163*P*)^2^ + 4.6062*P*] where *P* = (*F*_o_^2^ + 2*F*_c_^2^)/3
2376 reflections	(Δ/σ)_max_ = 0.001
154 parameters	Δρ_max_ = 1.10 e Å^−^^3^
0 restraints	Δρ_min_ = −0.44 e Å^−^^3^

### Special details

**Table:**

Geometry. All e.s.d.'s (except the e.s.d. in the dihedral angle between two l.s. planes) are estimated using the full covariance matrix. The cell e.s.d.'s are taken into account individually in the estimation of e.s.d.'s in distances, angles and torsion angles; correlations between e.s.d.'s in cell parameters are only used when they are defined by crystal symmetry. An approximate (isotropic) treatment of cell e.s.d.'s is used for estimating e.s.d.'s involving l.s. planes.
Refinement. Refinement of *F*^2^ against ALL reflections. The weighted *R*-factor *wR* and goodness of fit *S* are based on *F*^2^, conventional *R*-factors *R* are based on *F*, with *F* set to zero for negative *F*^2^. The threshold expression of *F*^2^ > σ(*F*^2^) is used only for calculating *R*-factors(gt) *etc*. and is not relevant to the choice of reflections for refinement. *R*-factors based on *F*^2^ are statistically about twice as large as those based on *F*, and *R*- factors based on ALL data will be even larger.

### Fractional atomic coordinates and isotropic or equivalent isotropic displacement parameters (Å^2^)

**Table:**

	*x*	*y*	*z*	*U*_iso_*/*U*_eq_	
C1	0.1795 (2)	0.2005 (4)	0.33571 (13)	0.0204 (6)	
C2	0.1524 (2)	0.1513 (4)	0.27097 (13)	0.0187 (6)	
C3	0.0612 (2)	0.1987 (5)	0.24969 (14)	0.0253 (7)	
H3	0.0170	0.2563	0.2759	0.030*	
C4	0.0363 (2)	0.1603 (5)	0.18965 (13)	0.0231 (7)	
H4	−0.0247	0.1919	0.1756	0.028*	
C5	0.1021 (2)	0.0744 (4)	0.15012 (12)	0.0148 (5)	
C6	0.1935 (2)	0.0297 (5)	0.17129 (14)	0.0223 (6)	
H6	0.2382	−0.0253	0.1449	0.027*	
C7	0.2183 (2)	0.0667 (5)	0.23162 (14)	0.0242 (7)	
H7	0.2792	0.0348	0.2457	0.029*	
C8	0.3754 (2)	0.5305 (4)	0.46896 (15)	0.0202 (6)	
C9	0.4829 (2)	0.5423 (5)	0.46975 (14)	0.0238 (7)	
H9A	0.5034	0.6766	0.4660	0.029*	
H9B	0.5095	0.4688	0.4358	0.029*	
C10	0.0752 (2)	0.0328 (4)	0.08488 (12)	0.0126 (5)	
O1	0.25854 (17)	0.1379 (4)	0.35567 (9)	0.0256 (5)	
O2	0.12255 (18)	0.3032 (3)	0.36600 (10)	0.0274 (5)	
O3	0.33421 (15)	0.3741 (3)	0.45414 (10)	0.0200 (4)	
O4	0.32547 (15)	0.6772 (3)	0.48303 (9)	0.0178 (4)	
O5	0.13755 (15)	−0.0398 (3)	0.05016 (9)	0.0173 (4)	
O6	−0.00860 (15)	0.0752 (3)	0.06791 (9)	0.0184 (4)	
Tb1	0.331426 (9)	0.019240 (18)	0.445275 (6)	0.01157 (5)	

### Atomic displacement parameters (Å^2^)

**Table:**

	*U*^11^	*U*^22^	*U*^33^	*U*^12^	*U*^13^	*U*^23^
C1	0.0303 (17)	0.0194 (14)	0.0114 (13)	−0.0055 (12)	−0.0067 (12)	−0.0008 (12)
C2	0.0266 (16)	0.0182 (14)	0.0113 (13)	0.0008 (12)	−0.0074 (12)	−0.0024 (11)
C3	0.0226 (16)	0.0388 (19)	0.0146 (14)	0.0067 (13)	−0.0006 (12)	−0.0092 (14)
C4	0.0151 (14)	0.0381 (18)	0.0162 (14)	0.0072 (13)	−0.0058 (12)	−0.0084 (13)
C5	0.0169 (13)	0.0180 (13)	0.0094 (12)	−0.0011 (11)	−0.0012 (10)	−0.0023 (11)
C6	0.0173 (14)	0.0318 (17)	0.0179 (15)	0.0030 (12)	−0.0059 (11)	−0.0072 (13)
C7	0.0241 (16)	0.0305 (17)	0.0180 (14)	0.0078 (13)	−0.0104 (12)	−0.0039 (13)
C8	0.0167 (14)	0.0154 (14)	0.0285 (16)	0.0013 (11)	−0.0058 (12)	−0.0014 (12)
C9	0.0238 (16)	0.0236 (16)	0.0240 (16)	−0.0028 (12)	−0.0007 (13)	0.0018 (13)
C10	0.0161 (13)	0.0135 (13)	0.0082 (12)	−0.0042 (10)	−0.0014 (10)	0.0017 (10)
O1	0.0288 (12)	0.0363 (13)	0.0116 (9)	−0.0006 (10)	−0.0090 (9)	0.0023 (9)
O2	0.0365 (14)	0.0298 (12)	0.0159 (10)	0.0018 (10)	−0.0071 (10)	−0.0108 (10)
O3	0.0136 (10)	0.0126 (9)	0.0338 (12)	0.0003 (8)	−0.0069 (9)	−0.0015 (9)
O4	0.0175 (10)	0.0150 (9)	0.0209 (10)	0.0014 (8)	−0.0025 (8)	−0.0017 (8)
O5	0.0184 (10)	0.0253 (11)	0.0083 (9)	0.0039 (8)	0.0010 (7)	0.0000 (8)
O6	0.0134 (10)	0.0294 (11)	0.0123 (9)	−0.0010 (9)	−0.0036 (8)	−0.0009 (8)
Tb1	0.01146 (7)	0.01367 (7)	0.00960 (7)	−0.00056 (5)	−0.00011 (5)	−0.00135 (5)

### Geometric parameters (Å, °)

**Table:**

C1—O2	1.252 (4)	C9—H9A	0.9700
C1—O1	1.261 (4)	C9—H9B	0.9700
C1—C2	1.503 (4)	C10—O5	1.257 (3)
C2—C7	1.386 (4)	C10—O6	1.260 (3)
C2—C3	1.393 (4)	O1—Tb1	2.352 (2)
C3—C4	1.382 (4)	O2—Tb1^i^	2.370 (2)
C3—H3	0.9300	O3—Tb1	2.447 (2)
C4—C5	1.391 (4)	O3—Tb1^i^	2.524 (2)
C4—H4	0.9300	O4—Tb1^iii^	2.492 (2)
C5—C6	1.392 (4)	O4—Tb1^i^	2.578 (2)
C5—C10	1.501 (4)	O5—Tb1^iv^	2.3359 (19)
C6—C7	1.386 (4)	O6—Tb1^v^	2.283 (2)
C6—H6	0.9300	Tb1—O6^vi^	2.283 (2)
C7—H7	0.9300	Tb1—O5^vii^	2.3359 (19)
C8—O3	1.261 (4)	Tb1—O2^viii^	2.370 (2)
C8—O4	1.263 (4)	Tb1—O4^ix^	2.492 (2)
C8—C9	1.501 (4)	Tb1—O3^viii^	2.524 (2)
C8—Tb1^i^	2.932 (3)	Tb1—O4^viii^	2.578 (2)
C9—C9^ii^	1.521 (6)	Tb1—C8^viii^	2.932 (3)
			
O2—C1—O1	124.4 (3)	C8—O4—Tb1^iii^	130.8 (2)
O2—C1—C2	117.7 (3)	C8—O4—Tb1^i^	93.10 (17)
O1—C1—C2	117.9 (3)	Tb1^iii^—O4—Tb1^i^	108.64 (7)
C7—C2—C3	119.8 (3)	C10—O5—Tb1^iv^	134.11 (18)
C7—C2—C1	120.7 (3)	C10—O6—Tb1^v^	154.76 (19)
C3—C2—C1	119.4 (3)	O6^vi^—Tb1—O5^vii^	86.13 (7)
C4—C3—C2	120.1 (3)	O6^vi^—Tb1—O1	105.02 (8)
C4—C3—H3	120.0	O5^vii^—Tb1—O1	151.11 (8)
C2—C3—H3	120.0	O6^vi^—Tb1—O2^viii^	75.44 (8)
C3—C4—C5	120.3 (3)	O5^vii^—Tb1—O2^viii^	134.79 (8)
C3—C4—H4	119.8	O1—Tb1—O2^viii^	74.10 (9)
C5—C4—H4	119.8	O6^vi^—Tb1—O3	80.00 (7)
C4—C5—C6	119.5 (3)	O5^vii^—Tb1—O3	81.91 (7)
C4—C5—C10	120.4 (3)	O1—Tb1—O3	74.17 (8)
C6—C5—C10	120.2 (3)	O2^viii^—Tb1—O3	132.69 (8)
C7—C6—C5	120.3 (3)	O6^vi^—Tb1—O4^ix^	103.49 (7)
C7—C6—H6	119.9	O5^vii^—Tb1—O4^ix^	74.84 (7)
C5—C6—H6	119.9	O1—Tb1—O4^ix^	126.02 (8)
C6—C7—C2	120.1 (3)	O2^viii^—Tb1—O4^ix^	70.12 (8)
C6—C7—H7	120.0	O3—Tb1—O4^ix^	156.11 (7)
C2—C7—H7	120.0	O6^vi^—Tb1—O3^viii^	166.21 (7)
O3—C8—O4	119.4 (3)	O5^vii^—Tb1—O3^viii^	96.79 (7)
O3—C8—C9	120.2 (3)	O1—Tb1—O3^viii^	78.76 (8)
O4—C8—C9	120.3 (3)	O2^viii^—Tb1—O3^viii^	93.23 (8)
O3—C8—Tb1^i^	58.93 (15)	O3—Tb1—O3^viii^	113.73 (5)
O4—C8—Tb1^i^	61.42 (15)	O4^ix^—Tb1—O3^viii^	64.62 (7)
C9—C8—Tb1^i^	170.4 (2)	O6^vi^—Tb1—O4^viii^	143.04 (7)
C8—C9—C9^ii^	107.6 (3)	O5^vii^—Tb1—O4^viii^	79.52 (7)
C8—C9—H9A	110.2	O1—Tb1—O4^viii^	75.72 (8)
C9^ii^—C9—H9A	110.2	O2^viii^—Tb1—O4^viii^	136.68 (8)
C8—C9—H9B	110.2	O3—Tb1—O4^viii^	64.43 (7)
C9^ii^—C9—H9B	110.2	O4^ix^—Tb1—O4^viii^	105.25 (6)
H9A—C9—H9B	108.5	O3^viii^—Tb1—O4^viii^	50.58 (6)
O5—C10—O6	123.8 (2)	O6^vi^—Tb1—C8^viii^	168.43 (8)
O5—C10—C5	118.4 (2)	O5^vii^—Tb1—C8^viii^	90.44 (8)
O6—C10—C5	117.8 (2)	O1—Tb1—C8^viii^	73.28 (9)
C1—O1—Tb1	141.6 (2)	O2^viii^—Tb1—C8^viii^	114.33 (9)
C1—O2—Tb1^i^	124.6 (2)	O3—Tb1—C8^viii^	88.58 (7)
C8—O3—Tb1	151.30 (19)	O4^ix^—Tb1—C8^viii^	86.20 (8)
C8—O3—Tb1^i^	95.72 (17)	O3^viii^—Tb1—C8^viii^	25.35 (7)
Tb1—O3—Tb1^i^	111.92 (8)	O4^viii^—Tb1—C8^viii^	25.48 (7)

Symmetry codes: (i) −*x*+1/2, *y*+1/2, *z*; (ii) −*x*+1, −*y*+1, −*z*+1; (iii) *x*, *y*+1, *z*; (iv) −*x*+1/2, −*y*, *z*−1/2; (v) *x*−1/2, *y*, −*z*+1/2; (vi) *x*+1/2, *y*, −*z*+1/2; (vii) −*x*+1/2, −*y*, *z*+1/2; (viii) −*x*+1/2, *y*−1/2, *z*; (ix) *x*, *y*−1, *z*.
